# Editorial: Food of the future: meat and dairy alternatives

**DOI:** 10.3389/fnut.2024.1382337

**Published:** 2024-02-27

**Authors:** Jean-François Hocquette, Sghaier Chriki, Marie-Pierre Ellies-Oury, Fang Fang, Antti Knaapila

**Affiliations:** ^1^INRAE, Clermont Auvergne, Université Clermont Auvergne, VetAgro Sup, UMR1213, Recherches sur les Herbivores, Saint Genès Champanelle, France; ^2^Isara, Lyon, France; ^3^Feed & Food Department, Bordeaux Sciences Agro, Gradignan, France; ^4^IEH Laboratories and Consulting Group, Lake Forest Park, WA, United States; ^5^Department of Food and Nutrition, University of Helsinki, Helsinki, Finland

**Keywords:** meat, dairy products, meat analogs, dairy analogs, dietary changes, future food

In recent years, there has been increasing discussion about the impact of our dietary choices not only on our health, but also on global issues such as food security and climate change. Much emphasis has been placed on eating a plant-based diet and avoiding foods of animal origin, with vegetarian options becoming far more abundant across grocery stores and in restaurants. But are we on the right direction? Will meat and dairy alternatives satisfy consumers by living up to their promises? To contribute to this debate, 11 articles have been published in this special issue including 5 on what is called “cultured meat,” 4 on plant-based meat/dairy alternatives, and 2 on “hybrid meat” or other alternatives.

In June 2023, the United States became the second country after Singapore to approve the commercialization of “cultured meat” despite uncertainties about this product (1). Failla et al. analyzed 1,151 comments submitted to the 2021 U.S. Department of Agriculture's Food Safety and Inspection Services (USDA-FSIS) call on the labeling of cell-cultured meat. Cultured meat was the preferred labeling term. The majority of comments came from people with unknown affiliation. However, many comments came from farmer advocacy groups and then cell-cultured meat companies. Comments from cell-cultured meat companies and animal welfare associations had the highest median word count. From a recent study, farmers do express complex and nuanced opinions related to food system control and transparency associated with cultured meat as well as potential impacts on the environment, the land, employment, and the life of farming/rural communities (2).

Most investment and research into cultured meat has so far occurred in the US. However, Attwood et al. argued that cultured meat is, so far, an untapped opportunity for the Muslim market thanks to the high projected increase in the world's Muslim population in Asia and Africa. Whether cultured meat can be certified as halal is therefore of paramount importance. Then the potential acceptance of cultured meat by Muslim consumers' needs to be studied in detail, taking into account their specific culture.

In South-Western Europe (Italy, Portugal, and Spain), Liu et al. observed a positive initial attitude toward cultured meat despite fragmented opinions. Indeed, almost two thirds of the respondents were willing to taste cultured meat but only 43% to eat it regularly and 94% would not pay more compared to conventional meat. Younger respondents, scientists or respondents unfamiliar with the meat sector had a higher acceptance. Ethical and environmental concerns were the major motives. Conversely, emotional resistance and lower perceptions of the benefits of cultured meat and of the weaknesses of conventional meat were the main barriers to acceptance of cultured meat.

Using the same survey in 12 African countries (Cameroon, Congo, -DRC Democratic Republic of Congo, Ghana, Ivory Coast, Kenya, Morocco, Nigeria, Senegal South Africa, Tanzania, and Tunisia), Kombolo Ngah et al. confirmed some previous observations, especially the low willingness to pay for cultured meat. Furthermore, people were more likely to try this novel food in the richest and most educated countries surveyed. In addition, a large proportion of respondents strongly agreed that cultured meat would have a negative impact on the rural life confirming other studies conducted using the same protocol but on the French population (3).

Cultured meat is also expected to meet consumers' wishes in terms of sensory and nutritional value, which is not the case yet according to Fraeye et al. (4) and Olenic and Thorrez (1). To et al. analyzed 26 studies directly related to the sensory evaluation of cultured meat. Despite bias due to some potential conflicts of interest for many authors, To et al. attempted to distinguish between what is actually known and all the speculation in order to identify real expectations regarding the sensory characteristics of cultured meat, given the promising narratives of all the proponents.

The lack of standardized terminology for non-animal-based alternatives to animal-based foods has led to the interchangeable use of terms such as meat substitute, replacement, and analog. Addressing this ambiguity, Abbaspour et al. propose a welcome classification. They define “substitute” as a similar product from a culinary perspective, emphasizing functional and sensory properties. “Replacement” refers to options with similar nutritional properties. “Analog” seeks to match both culinary and nutritional attributes, while “alternative” represents a different choice, not necessarily mirroring the original product.

In a broader context, products derived from gene-edited farm animals could also be considered alternatives to conventional animal-based foods. In their study, Martin-Collado et al. explored societal attitudes toward gene-edited meat products. The findings revealed that consumers perceive gene-edited foods akin to genetically modified foods. The authors emphasized the importance of ongoing dialogue to inform consumers about this innovative technology.

Hybrid meat, combining both animal and plant-based proteins, has been observed to face a challenge in consumer acceptance due to limited familiarity (5). In a study by Ryder et al., consumers' verbal associations with hybrid meat improved after a co-creation task, demonstrating positive shifts as familiarity and ingredient knowledge increased. The research underscores a significant obstacle: consumers' lack of understanding regarding the nature and processes involved in developing hybrid meat products.

In their study, Kuosmanen et al. investigated consumers' perceived barriers associated with consumption of selected plant-based alternatives to meat (pulses and meat analogs). The authors employed the Capability, Opportunity, Motivation, Behavior (COM-B) model to interpret the results and observed that the most common perceived barriers for the consumption were unfamiliarity (capability), expensive price (opportunity), and unpleasant taste (motivation).

In addition to meat alternatives, dairy alternatives, such as plant-based milks and yogurts, are actively under study. In the research of D'Andrea et al., the nutritional value of plant-based yogurts was compared to that of dairy yogurts in the US market. The findings revealed that plant-based yogurts contained lower amounts of sugars and sodium, and more fiber, but less protein, calcium, and potassium compared to their dairy counterparts. This study underscores the variability in nutritional profiles between animal-based products and their plant-based alternatives.

McCarron et al. studied oat-based milk alternatives commercially available in the UK. The results indicated that achieving a small particle size is a key target feature, as it correlates with increased lightness, reduced perception of off-white color, and a diminished powdery mouthfeel. Furthermore, the findings suggested avoiding clear (transparent) packaging to prevent off-notes resulting from photo-oxidation. The study emphasizes the importance of sensory analysis in the development of new products.

The articles featured in this Research Topic illustrate that the partial evolution from animal-based to non-animal-based foods not only presents technological challenges but also demands considerations for integrating the alternatives into consumers' diets. Ensuring availability, an acceptable price, and attractive sensory properties are crucial aspects in addition to ethical and environmental benefits. Furthermore, it is essential to address consumer unfamiliarity through objective information based on scientific facts about ingredients, production processes, and nutritional values, as well as offering guidance on preparation. We hope this collection of articles provides insights that inspire further research on the topic.

## Author contributions

J-FH: Writing—original draft, Writing—review & editing. SC: Writing—original draft, Writing—review & editing. M-PE-O: Writing—original draft, Writing—review & editing. FF: Writing—original draft, Writing—review & editing. AK: Writing—original draft, Writing—review & editing.
